# Utilization of Ground Eggshell as a Biofiller of Plasticized PVC-Based Materials Fabricated Using Melt Blending

**DOI:** 10.3390/polym17040434

**Published:** 2025-02-07

**Authors:** Katarzyna Skórczewska, Krzysztof Lewandowski, Sławomir Wilczewski, Joanna Szulc, Paulina Rakowska

**Affiliations:** Faculty of Chemical Technology and Engineering, Bydgoszcz University of Science and Technology, Seminaryjna 3, 85-326 Bydgoszcz, Poland; krzysztof.lewandowski@pbs.edu.pl (K.L.); joanna.szulc@pbs.edu.pl (J.S.); paulina_rakowska93@wp.pl (P.R.)

**Keywords:** eggshells, composites, ecofriendly filler, modification, properties, thermal properties

## Abstract

The paper examines the use of waste eggshells as a valuable biofiller for modifying plasticized poly(vinyl chloride) (PVC). The raw ES was characterized using TGA, FTIR, particle size analysis, and XRD. The effects of ES on the processing, mechanical and thermal properties, density, porosity, and colour of PVC matrix composites were evaluated compared to pPVC/CC produced using the same methodology. It was found that pPVC/ES exhibits different processing properties to pPVC/CC. The mechanical properties of PVC/ES are slightly lower than those of pPVC/CC at concentrations up to 20 phr. However, at 30 phr and 40 phr, the differences in the mechanical properties of composites with both CC and ES are very similar, and the values are within the designated standard deviation of the measurement. The mechanical properties of PVC/ES do not limit their potential applications. When using eggshell (ES) as a filler, improvements in tensile strength (t_ts_) were observed, ranging from 38% to 61% compared to the unfilled matrix and from 35% to 54% compared to pPVC/CC with an equivalent amount of filler. Although ground eggshells have similar insulating properties to calcium carbonate (CC), they are more effective at scavenging chlorine (Cl•) released during the initial stages of decomposition. This effectiveness helps to slow down the breakdown of PVC, as the eggshells maintain their porous, sponge-like structure when used as a filler.

## 1. Introduction

The increasing demand for food is also causing an increase in food processing waste and its tendency to accumulate in the environment. One common food waste is eggshells, especially chicken eggs. In addition, the problem of microbiological hazards is increasing with this waste, namely due to the presence of organic matter; this waste can cause microbiological hazards and can also be a source of pathogenic contamination [1,2]. For these reasons, European Union legislation treats this eggshell waste as hazardous waste [3,4,5,6]. However, many reports indicate that it may be a material with highly desirable properties and valuable applications [2,7,8] and thus ideally suited to a zero waste economy.

In 2022, approximately 87 Mt of chicken eggs were produced worldwide and 6.3 Mt of eggs in the European Union [9]; taking into account that eggshells make up on average between 9% [10] and 11% of an egg’s weight [11], the amount of waste generated (assuming 11%) is 9.6 Mt worldwide and 697,925.5 tonnes in the EU, respectively. Trends indicate a significant increase in the demand for this food product and a successive increase in its production and thus in waste. Eggshell waste as an unnecessary raw material usually ends up in landfills without any transformation, but given the guidelines of a sustainable economy based on the recycling and reuse of materials, this undervalued material should be considered for reuse.

There are an increasing number of reports in the literature on the reuse of eggshell waste as a source of collagen, hyaluric acid [12,13], hydroxyapatite [14,15], biotechnological catalysts, and absorbents [14], as a valuable functional food additive affecting its health-promoting properties [16], or for use in modern ceramic materials. Eggshells, in particular, are also being intensively researched for use as a modifier of polymer materials [11,15,16,17,18,19,20,21,22,23,24,25].

Poly(vinyl chloride) (PVC), due to its favourable performance properties and its ability to be modified over a wide range of properties, as well as its competitiveness in terms of price, is a polymer with a wide range of applications, from construction applications, which require good mechanical characteristics and stable properties over a long lifetime, to medical applications requiring biocompatibility. The rigid varieties of this polymer are used for sewerage pipes, water pipes, furniture profiles, window profiles, floorboards, or drug blister packs. The plasticized, flexible varieties containing varying proportions of plasticizers are used for cable insulation, films, floor coverings, roof coverings, hoses, and packaging films. This polymer requires the application of thermal stabilizers during processing. Therefore, it is crucial to develop a material formulation with improved thermal stability [26]. It is also common to introduce mineral fillers into the PVC matrix in the form of calcium carbonate, titanium oxide, silica, clay minerals, talc, ground dolomite (calcium magnesium carbonate) and limestone fillers magnesium and aluminum hydroxides, and carbon black. This common practice of modifying PVC in various ways, mainly through fillers, is so widespread that it leads to a material with properties tailored to the individual requirements and needs of the user.

One of the most commonly used fillers for PVC, in both rigid and flexible grades, is calcium carbonate [27], which is derived from mined calcite mineral, which is ground to a suitable size (GCC—ground calcium carbonate) and then surface-treated with, e.g., stearate derivatives. This filler can be obtained through calcination, hydration, recarbonation, and precipitation from solutions as synthetically precipitated calcium carbonate (PCC), leading to a high-purity, size-controlled material [28]. The use of inorganic additives such as calcium carbonate (CaCO_3_) in the PVC blend is a well-known approach to improve PVC’s thermo-mechanical, fire, and light resistance properties, as well as to reduce the emission of hazardous substances resulting from the thermal degradation of PVC. Given the volumes of eggs produced, eggshell waste can be a valuable and low-cost source of sustainable biobased filler.

Adding CaCO_3_ to plasticized PVC changes its mechanical properties, mainly to increase its hardness, dimensional stability, and density. A significant proportion of chalk in the matrix can absorb the plasticizer introduced into PVC, causing a significant reduction in the elongation value at the break. It is also used as an additive to reduce the initial migration of the plasticizer [27]. Chalk also influences the colour and surface appearance of the product by giving it a smooth finish. The addition of CaCO_3_, due to its low price, can reduce the cost of the final product. It also has a weak stabilizing effect when processing PVC blends at higher temperatures, lowers the LOI, and reduces smoke generation [29,30].

The eggshell is a porous calcite biostructure with a protective function, enabling gas exchange. The shell contains CaCO_3_ as the main component, accounting for 92–97% of the mass [31], which imparts hardness and strength. It also contains about 5% organic substances in the forms of the inner membrane, cuticle, or amino acids of collagen. In addition, the eggshell may contain small amounts of MgO P_2_O_5_,Al_2_O_3_, SiO_2_ (0.07%), Cl_2_O_3_, and SrO [32].

The CaCO_3_ structures in the shell are organized on the inside with a mammillary layer, with a regular arrangement of cones or nodules on which calcite microcrystals with spherulitic texture are deposited. Their columnar perpendicular arrangement to the crust’s surface forms a palisade layer; on the outer surface of the shell, there is a more compact and stronger layer of calcite [6]. This highly porous structure, where pores with a three-level hierarchy, nano-scale pores, sub-micro-scale pores (200–400 nm) [33] (‘bubble pores’), and micro-scale pores, are distributed in the palisade and mammillary layers [34], ensures gas exchange with the embryo.

A natural destination for waste eggshells consisting of 94–95% CaCO_3_ [35] could also be their use as a source of biobased filler for PVC modification, where CaCO_3_ is commonly used. Despite the growing interest in using ES as an environmentally friendly biobased filler for polymeric plastics, unfortunately, little information is available on PVC matrix composites [36,37,38,39,40,41,42]. These reports concern unplasticized PVC composites or composites obtained by solution casting and solvent evaporation. Kiryakova D et al., in [36,41], investigated the effect of the surface modification of ES filler by vinyltrimethoxysilane surface treatment on the properties of PVC composites obtained by solution pouring and then evaluated their mechanical properties before and after immersion in water for 25 days, confirming the beneficial effect of surface modification with silane. In another paper [41], Kiryakova D. et al. described the use of silane to modify PVC composites obtained by the co-precipitation of solutions from cyclohexanone. In our earlier work [37], we characterized unplasticized PVC composites containing up to 40 phr of filler produced by extrusion, in which we indicated the filler’s positive effect on thermal stability and softening temperature. Murugan S. [40] investigated the effect of eggshell filler particle size on the properties of plasticized PVC, finding slightly more favourable mechanical properties of composites using particles of a 2-micrometre size compared to composites modified with particles of a 7-micrometre size. M. Sharmeeni et al. [39] studied the effect of the sequence of mixing the components of PVC composition with dioctylephthalate (DOP) plasticizer (30 wt%), ES filler, and process additives on the processability and properties of the obtained material. Different material properties were obtained using different component mixing procedures, i.e., mixing PVC with plasticizer and introducing ES filler (1) or combining PVC with ES filler initially mixed with plasticizer (2). Comparing the manufacturing procedures, the materials differed in their mechanical properties, especially at 30 and 40 wt% ES filler content, which, according to the authors of this work, is due to plasticizer absorption by the porous mineral filler and, thus, its reduced concentration in the matrix. In the industrial production of highly plasticized and highly filled PVC matrix materials, to avoid the effect of plasticizer absorption by the mineral filler, PVC is first mixed with plasticizer, and then, after its absorption by the PVC grains, the mineral filler is introduced. In contrast, a study [42] has described the effectiveness of solvent-free metallic soaps derived from naturally occurring epoxidized vegetable oils and biogenic calcium carbonate derived from eggshells in the context of the use of PVC plastic artificial leather with lower volatile organic compound (VOC) emissions.

To date, no comparative study has been reported in the literature on the properties of PVC composites plasticized with naturally derived epoxidized soybean oil [43] and modified with waste eggshells or chalk while maintaining the same processing methodology. Furthermore, the literature does not compare colour changes in PVC composites containing ES filler. In the presented work, the biofiller produced by milling was intentionally used as an unmodified filler to determine the level of its influence on the properties of PVC composites. The presented research will be the basis for the further development of soft PVC/ES composites, if only in the direction of future modifications and the elimination of imperfections of the used filler. The prepared soft PVC/ES material, characterized by favourable properties, is part of the concept of more environmentally friendly materials by reusing waste material of natural origin.

## 2. Materials and Methods

### 2.1. Materials

A dry blend of suspension PVC was used to produce the plasticized composite: suspension PVC type Neralit S-601 (Spolana s. r. o., Neratovice, Czech Republic)—100 phr; organotin stabilizer Patstab 2310 (Patcham, Holten, The Netherlands)—2 phr; Lubricant Ceasit (Baerlocher Production, Cincinnati, OH, USA)—1.2 phr; the paraffin wax Naftolube FTP—0.5 phr (Chemson, Arnoldstein, Austria); Loxiol G-32 (Emery Oleochemicals, Cincinnati, OH, USA)—1.5 phr; Paraloid K-125—1 phr; and Paraloid K-175—1 phr (both from Dow Chemical Company, Midland, MI, USA).

The Ergoplast ES (EOS), epoxidized soybean oil purchased from Boryszew-Erg S.A. (Sochaczew, Poland), with a maximum density of 0.998 g/cm3, was used as a plasticizer.

Waste eggshells from the Rosa hen breed obtained from a small farm (Mała Cerkwica, Poland) were used to produce a biofiller (ES) to modify plasticized PVC. The same filler source was used to obtain a filler to modify unplasticized PVC, as described in our previous work [37].

To compare the effect of the resulting nanofiller on the properties of the plasticized PVC composites, Omya Chalk 2T-VA (CC) (Omya Sp. z o.o, Warsaw, Poland) was applied. This marble chalk from Vápenná (Czech Republic) contains 98% CaCO_3_, is surface-modified, and is used to modify both rigid and plasticized PVC and polyolefins [44].

### 2.2. Eggshell Filler (ES) Preparation

The first step in producing the filler from waste eggshells was to separate the organic residues and protein membranes by separation in water and washing the crushed shells. To do this, the eggshells were poured with water and left to stand for 24 h. Then, they were washed several times until the protein membrane separation disappeared. The eggshells were then dried for 24 h at 80 °C and, after this time, ground with a ZM200 Retsch ultracentrifugal mill (Haan, Germany) and sieved with a vibratory sieve shaker ANALYSETTE 3 PRO, Fritsch (Idar-Oberstein, Germany), to obtain filler ES with a particle size smaller than 120 μm.

### 2.3. Materials Processing

The manufacturing of plasticized PVC (pPVC) composites was carried out in 2 main stages. In the first stage, an appropriate amount of dry blend PVC was introduced into the mixing chamber of a Z-shape blade mixer (FDO 234H, Brabender GmbH & Co. KG, Duisburg, Germany) heated to 90 °C. An appropriate amount of plasticizer was added and mixed for 10 min at 56 min^−1^. The plasticizer content was 30 phr (part per hundred), regardless of the composition. The resulting mixtures were left for 24 h at room temperature until the plasticizer was completely absorbed and a dry mixture was obtained.

An appropriate amount of ES was then added to the PVC mixtures and mixed using an Ika Eurostar 6000 high-speed mixer for 10 min at 2000 min^−1^. The homogeneous mixtures obtained were processed in a Brabender’s torque rheometer according to the procedure for the processing tests described below in Section 2.4.1. After cooling, the material obtained was ground with a grinder. The resulting grinding material was compressed using a hydraulic press at 185 °C for 5 min and at 10 MPa into 120 × 120 × 2 mm plates, from which test specimens were cut. The amount of ES in the composites was 0, 10 phr, 20 phr, 30 phr, and 40 phr, respectively. The samples were designated; for example, as pPVC/30ES-plasticized PVC (30 phr of EOS) containing 30 phr of ES.

According to the presented methodology, composites of plasticized PVC with calcium carbonate (CC) labelled as pPVC/CC were produced.

### 2.4. Testing Methods of ES Filler and pPVC Composites

#### 2.4.1. ES Analysis

An infrared spectroscopy study was carried out to compare CC and ES chemically. An Alpha instrument (Bruker Optics GmbH & Co. KG, Ettlingen, Germany) was used, using the ATR (reflectance) technique, in the range of 4000–500 cm^−1^, with 32 scans at a resolution of 4 cm^−1^.

The particle size distribution of the produced filler was evaluated using a laser particle sizer Fritsch ANALYSETTE 22 (Idar-Oberstein, Germany) apparatus working in the range of 0.08–2000 μm.

The thermal stability of the ES was assessed using a thermogravimetric method in accordance with the polymer material testing methodology outlined below.

To assess the structure of the ES filler produced compared to CC, a wide-angle X-ray diffraction study was carried out using an X-ray URD 6 diffractometer from Rich Seifert & Co GmbH (Freiberg, Germany). Monochromatic X-ray diffraction with a wavelength of λ = 1.5406 Å (CuKα) in the 2θ angle range from 10 to 70 ° with step 0.05 was used.

#### 2.4.2. Methodology for Testing pPVC and Composites

The processing properties of the prepared PVC mixtures were determined by a plastographometric method using an FDO 234H torque rheometer (Brabender GmbH & Co., Duisburg, Germany) equipped with a 50 cm^3^ chamber. The measurement was performed at 185 °C and a rotational speed of the slower rotor of 30 min^−1^, using a charge mass of 60 g. The measurement was conducted for 10 min, recording the torque and mass temperature change as a function of the kneading time [45,46]. From the plastograms obtained, the characteristic torque (M) and temperature (T) values were determined after the respective kneading times, i.e., after 2 min, 5 min, and 10 min.

The colour of the PVC materials was evaluated according to the guidelines of the Commission Internationale de l’Eclairage (CIE) by measuring the L*a*b* colour coordinates. L* (brightness), a* (red–green), and b* (yellow–blue) were recorded using a colorimetric spectrophotometer (Chroma Meter CR-410, Tokyo, Japan), where the illuminant and reference angle were D65 and 2°, respectively. The measurement was performed at ten sample locations with three repetitions, and the values are presented as arithmetic averages of the result. The total colour difference parameter (ΔE), defined according to Formula (1), was also determined [47]:

(1) $$\Delta E=[({\Delta L}^{*}{)}^{2}+({\Delta a}^{*}{)}^{2}+({\Delta b}^{*}{)}^{2}{]}^{0.5}$$

The specified colour parameters in the L*a*b* space were used to convert to the Adobe RGB space, according to the methodology presented in the [48].

The material density was measured with a Pycnomatic gas pycnometer by Thermo Fisher Scientific Inc. (Waltham, MA, USA) using helium at a pressure of 0.2 MPa at 20 °C and employing a 40 cm^3^ measuring cell. The theoretical density was determined based on Equation (1):

(2) $$\rho_{th}=\rho_{m}\left(1-\varphi \right)+\rho_{f}\times \varphi$$

where $\rho_{th}$—theoretical density of the composite, g/cm^3^; $\rho_{m}$—density of the matrix, g/cm^3^; $\rho_{f}$—density of the filler, g/cm^3^; and φ—a volume fraction of the filler.

Then, the porosity of the materials was determined according to Equation (3).

(3) $$p=\frac{\rho_{th}-\rho_{ex}}{\rho_{th}}\times 100\%$$

where p—porosity, % and ρ_ex_—an experimental density of composite, g/cm^3^ [49].

The mechanical properties in the static tensile test were determined in accordance with EN ISO 527 [50]. The specimens used were (type 5 B), cut from pressed plates with dimensions of 120 mm × 120 mm × 2 mm using a standardized punch. Measurements were performed using a Zwick/Rolel Z010 universal testing machine (Zwick GmbH & Co., KG, Ulm, Germany) at 23 °C. The test speed was 100 mm/min. The maximum stress (σ_M_) and elongation at break (ε_B_) were determined. No fewer than 5 samples of each material type were tested. The hardness of the polymeric materials was determined by the Shore D method using a Zwick/Roell hardness tester.

The structure of the materials’ surfaces after mechanical testing was assessed by optical microscopy using a Nikon ECLIPSE E 400 POL microscope (Nikon, Tokyo, Japan). The microscope has a camera for continuous recording and NIS-Elecments 4.0 software. Imaging of the samples was performed in the reflected light.

The effect of ES and CC on the thermal stability of the composites was assessed by determining the time of thermal stability and by thermogravimetric tests.

The Congo red method was used to determine the time of thermal stability (t_ts_). The principle of the measurement is to determine the time after which hydrogen chloride is released from a sample of PVC material. A sample of the material was placed in a glass tube, a Congo red indicator paper was placed at the tube’s outlet, and the whole was placed in an oil bath heated to 200 °C. The measurement result is the time after which the first visible change in colour of the indicator paper is observed. Each material was measured three times.

The Determination of Thermal Properties by Thermogravimetric Analysis (TGA) was carried out in an oxidizing atmosphere using synthetic air as the gas. The measurement was carried out with a TG 209 F3 (Netzsch GmbH & Co. Holding KG, Selb, Germany) in the temperature range 30–900 °C with a temperature rate of 10 °C/min using samples of 15–20 mg and an air flow of 50 mL/min.

## 3. Results

### 3.1. FTIR Analysis of ES

**Figure 1:**
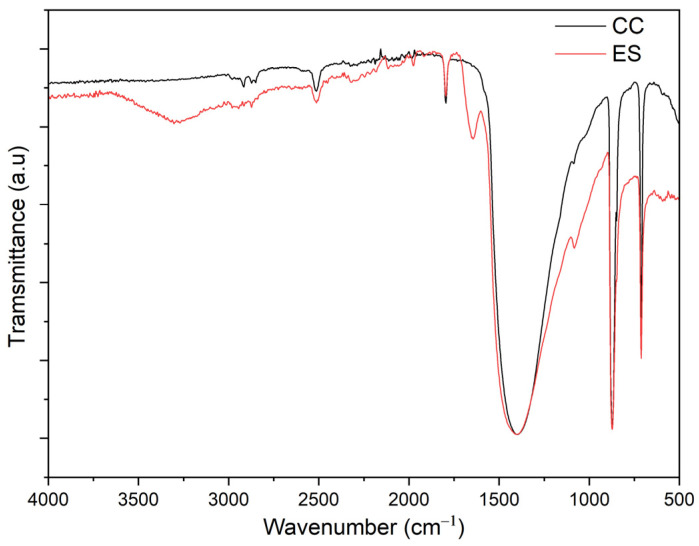
FTIR spectra of CC and ES (CC—black curve; ES—red curve).

Figure 1 shows the FTIR spectra of the chalk used (CC) and the ES produced from waste eggshells.

On both spectra of the fillers, some bands confirm the presence of CaCO_3_. On the FTIR ES spectrum, additional peaks, absent in the chalk, can be observed, i.e., 1645 cm^−1^ and 3419 cm^−1^. The peak at 1645 cm^−1^ is associated with the protein amide I group present in the ES and is indicative of the organic residue of the cuticle. The cuticle’s organic part comprises 85 to 90% protein, polysaccharides, lipids, and pigment eggs [51]. In the form of a fatty acids group, this residue can have a compatibilist effect between the filler and the PVC matrix [52]. Therefore, the ES was not further modified, as is the practice with chalk. The FTIR spectrum of the ES additionally exhibits a broad band from 3700 to 2500 cm^−1^, associated with OH and amide I groups from water and proteins. A broad peak centred at about 1400 cm^−1^ on the FTIR spectra of both filler types is associated with carbonate groups from calcite crystals. Peaks at 873 cm^−1^, 712 cm^−1^, and 1795 cm^−1^ are also associated with CaCO_3_ [52,53]. The bands at 2919 and 2872 cm^−1^ represent C-H vibrations, which, in the case of ES, indicate the existence of organic residues in the form of lipids or fatty acids group, and in the case of chalk, indicate that the filler has undergone surface modification, e.g., stearic acid to facilitate miscibility and compatibilization with the PVC matrix [54].

### 3.2. Particle Size Analysis of ES

The obtained ES was characterized by an irregular and porous structure with sharp edges, as shown in more detail on SEM images in our previous work [37].

According to the technical data sheet, the chalk (CC) used is characterized by an upper particle diameter (D98%) of 12 μm, an average particle size (D50%) of 2.5 μm, a particle share < 2 μm of 43%, and a BET surface area of 3.8 m^2^/g [55]. In contrast, the ES produced was characterized by an upper particle diameter (D98%) of 120 μm, an average particle size (D50%) of 31 μm, and a particle size share of <2 μm of 4.6%. According to data from the literature, the BET value of the ES prepared in this way can be taken as being between 0.5 m^2^/g [56] and 0.56 m^2^/g [57]. The size of the ES is significantly higher than the CC due to the available industrial technology for its manufacture. However, it was concluded that, despite these significant differences in filler particle size, ES effect studies would be performed comparatively to the chalk commercially used in PVC composites, and if a positive effect of the ES on the pPVC matrix was obtained, the filler would be adjusted to commercial size in the following research step.

### 3.3. TGA Analysis of ES

**Figure 2:**
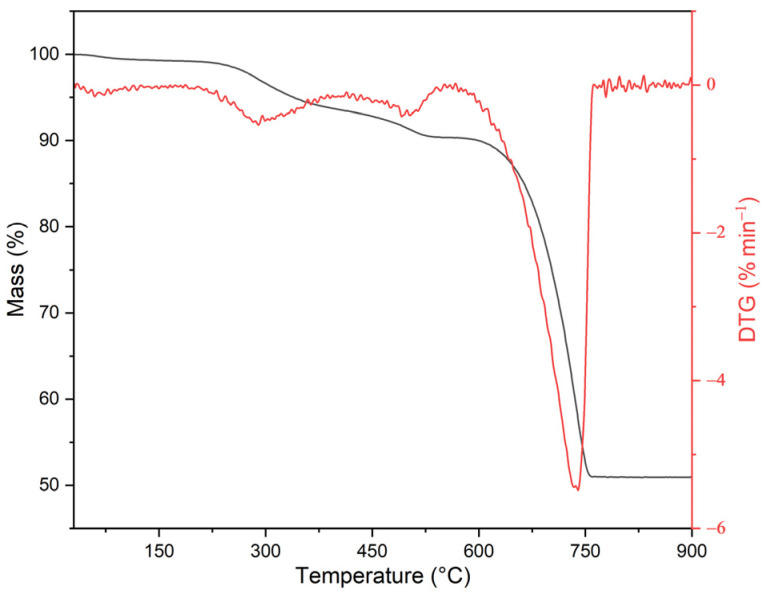
Thermogram TGA and DTG of ES (black curve—TGA; red curve—DTG).

The thermogravimetric analysis (Figure 2) assessed the possibility of using the resulting ES to modify plasticized PVC in terms of its thermal stability.

The procedure used to obtain the biofiller allowed the removal of residual water and the organic part with lower thermal stability, which could degrade during composite processing (up to 185 °C, the filler loses 0.7% of its initial mass). A loss of 1 wt.% by weight of the sample was recorded at 228 °C, i.e., higher than the processing temperature of the PVC. The first stage of decomposition to about 100 °C is associated with the evaporation of physically absorbed water. The next stage of decomposition to about 340 °C is associated with the decomposition of the organic part of the ES [58]. A further mass loss to about 540 °C is associated with the loss of dehydrated water in the lattice [59]. The decomposition of CaCO_3_ as the main component of ES occurs in the temperature range 650–760 °C with a maximum of 740 °C, resulting in the formation of CaO and the release of CO_2_ [3]. The estimated value for the proportion of CaCO_3_ in the eggshell was about 91%, which depends on how the hens are fed, which agrees with the results presented in [60].

### 3.4. XRD Results

**Figure 3:**
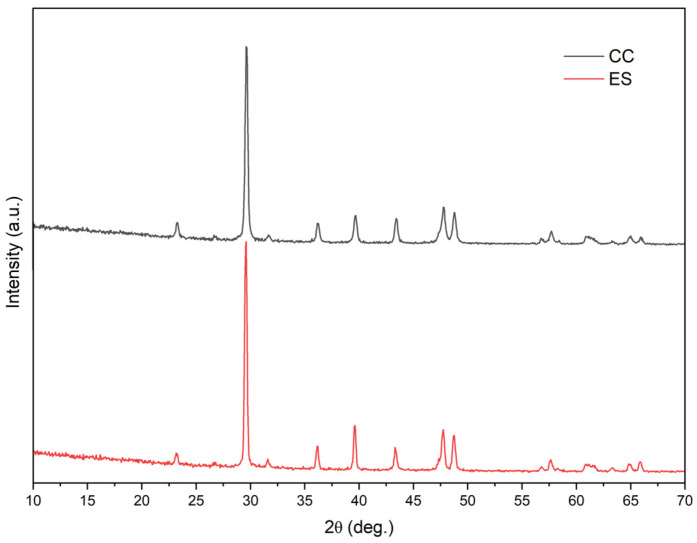
XRD patterns of ES and CC used fillers.

XRD studies indicate that the ES and CC fillers are characterized by a similar crystal structure (Figure 3), as evidenced by convergent crystal peaks in both spectra, which are characteristic of the calcite structure. The XRD peaks for the calcite appeared at approximately 2θ angles on 23.3°, 29.6°, 36.3°, 39.6°, 44.4°, 47.6°, 48.7°, and 57·6°. These results are consistent with the observations reported in the paper [61,62,63]. ES filler was created from grinding eggshells, the internal as well as external structure of which is slightly morphologically different, which is related to the presence of two essential layers of calcite which are structurally very different—the mammillary layer and palisade layer [64,65,66].

### 3.5. Plastographometric Analysis of pPVC and Composites

**Figure 4:**
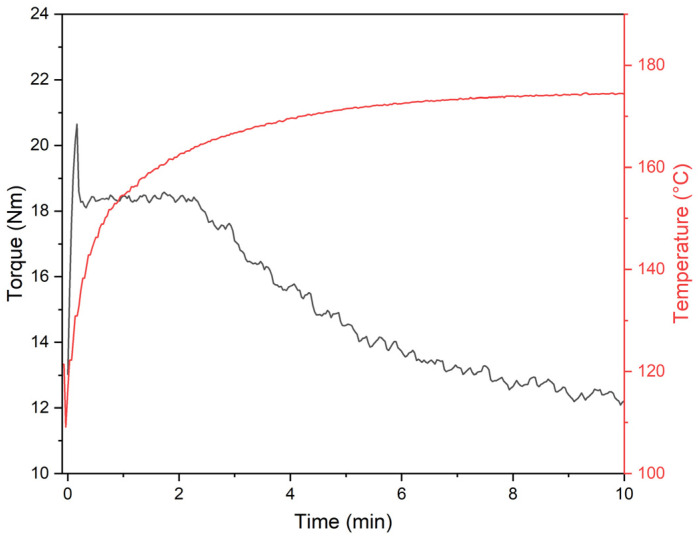
An exemplary plastogram of pPVC kneading (temperature—red curve; torque—black curve).

Processability analysis is used to assess the characteristics of the manufacturing process of composite materials and to adjust the relevant parameters of the process conducted, e.g., by changing the mixture components or processing parameters. Therefore, to compare the influence of ES on the processing properties of plasticized PVC blends, a plastographometric study was carried out [67,68] while measuring the change in the torque and temperature of the kneaded PVC blend as a function of time. The disintegration of the PVC grains and their melting occurs during this process. Additives introduced into PVC blends have a significant effect on this processing. In the case of plasticized blends, this process takes place at lower temperatures and lower torques compared to rigid PVC. The addition of fillers can accelerate or retard this process and also influence the torque and temperature. Figure 4 shows an example of a plastogram of a kneaded mixture of plasticized PVC. At the same time, Table 1 summarizes the characteristic values of temperature and torque evaluated from the plastograms at 2 min, 5 min, and 10 min of kneading PVC mixtures containing CC and ES.

The plastograms of the analyzed compositions are similar to those obtained by kneading an unfilled PVC matrix. The melting of the plasticized PVC grains and their gelation occur immediately after loading into the plastograph chamber. The torque value, over the entire test time range, of pPVC/CC blends is lower than that of pPVC at similar material temperatures. An increase in filler content usually leads to an increase in material viscosity, which should be followed by an increase in torque [69]. The different observations are probably due to two effects. The first is due to the change in the density of the material being processed, which, in the case of a constant mass measurement methodology, leads to a lower filling of the plastograph chamber and, therefore, a lower measured torque than would result from the viscosity of the composite. The CC particles are much smaller and similar in shape, so the heat is spread uniformly in the mass of the kneaded polymer material [70]. In addition, small particles of filler can exhibit an effect similar to that of external lubricants, increasing slip between the material and the processing equipment—in this case, the chamber wall and the surface of the rotating rotors [71,72]. This effect can be further enhanced by the CC surface modifiers used [73,74]

pPVC/ES composites exhibit different processing properties from pPVC/CC composites. The material heats up significantly slower during processing. Still, in this case, the microporous structure of ES can exhibit an insulating effect by impeding the heating of the processed material. Larger ES particle sizes and organic residues do not demonstrate the external lubricating effect observed in PVC/CC composites.

### 3.6. Chromatic Properties of Fillers and Polymeric Materials

The results of colourimetric tests of ES, CC, and polymeric materials in the CIE L*a*b* colour space and their conversion to the Adobe RGB space are summarized in Table 2. Table 2 also summarizes the ΔE value for the unfilled plasticized PVC matrix and the ΔE(CC:ES) value as the colour change for composites with different filler types but equal filler content and fillers alone. The coordinates a* and b* indicate the colour variation from red to green and from yellow to blue. L*, conversely, indicates variations in lightness (black—white). ΔE indicates colour differences and is associated with the human ability to distinguish colours and notice the colour difference, where the observer

- 0 < ΔE < 1—does not notice the difference,
- 1 < ΔE < 2—only an experienced observer will notice the difference,
- 2 < ΔE < 3.5—the inexperienced observer also notices the difference,
- 3.5 < ΔE < 5—notices a clear colour difference,
- 5 < ΔE—the observer gets the impression of two different colours.

**Table 2:** Colour properties of fillers and pPVC materials.

Sample	Colour Parameters					
	L*	a*	b*	ΔE	ΔE (ES:CC)	Colour
ES	89.07	0.74	6.23	-		
CC	95.13	0.14	4.12	-		
pPVC	74.64	0.84	33.51	0		
pPVC/10ES	72.48	2.77	25.93	8.1	4.5	
pPVC/20ES	73.02	2.99	23.13	10.7	5.4	
pPVC/30ES	71.53	3.00	21.67	12.4	7.4	
pPVC/40ES	71.57	3.04	20.79	13.3	6.0	
pPVC/10CC	76.57	1.15	26.60	7.2	4.5	
pPVC/20CC	76.64	0.81	19.82	13.8	5.4	
pPVC/30CC	77.01	0.55	17.28	16.4	7.4	
pPVC/40CC	75.45	0.45	16.97	16.6	6.0	

ΔE colour difference with target sample pPVC. ΔE (ES:CC) colour difference between samples with the same concertation of different filler.

CaCO_3_ is used in some applications as scattering pigments to ensure better whiteness [75,76,77,78]. Therefore, colour change studies were performed comparatively, analyzing the differences between PVC/CC and PVC/ES composites.

The pPVC sample was characterized by a colour close to yellow, which was due to the natural colour of the epoxidized soybean oil used as a plasticizer [79]. The analysis was therefore performed comparatively against this material. Compared to the unfilled matrix, the pPVC/CC composites are characterized by a higher L* value and a lower a* and b*. In contrast, pPVC/ES composites are characterized by a lower L* and b* value and a higher a*, i.e., the addition of ES results in a darker impression, a slight reduction in yellow, and a higher proportion of red. This could mainly be because of the intrinsic colour of the eggshell filler. Compared to PVC/CC composites, ES-containing materials have a lower L* value, indicating a darker colour; a higher a* (+) value, indicating a higher contribution of red; and a higher b* (+) value, indicating a higher contribution of yellow.

The calculated colour differences between the composites with different filler types and equal proportions of ΔE (ES:CC) and the fillers’ colour indicate a significant colour difference between the materials. ESs were less bright than the commercial CaCO_3_, due to the presence of eggshell pigments protoporphyrin and biliverdins [80,81]. Similar conclusions regarding colour change were reported for LDPE composites modified with eggshell filler, where the addition of ES resulted in the darkening of the composite colour (decreased the whiteness) [18], which may be due to Maillard reactions between residual protein substances contained in ES (FTIR studies indicated their presence) during composites processing.

The material with CC is lighter than the composite with ES, but this should not affect the possibility of using the composite with ES in the future due to the induced impression of a darker colour than the composite with CC. It seems that, as with composites with CC, in cases where the colour of the material or detail will be important, colourants could be added to adjust the appearance of pPVC/ES composites to desired values.

### 3.7. Density and Porosity

Table 3 summarizes the density values determined by the psychometric method and the porosity value calculated from Equation (3).

The ES has a lower density than chalk (by 15%); the density of ES was 2.27 g/cm^3^, while that of chalk was 2.67 g/cm^3^, which may be due to the more porous structure of eggshells, where pores are organized in a three-level hierarchy from nano pores to mesopores and micro pores. Plasticized PVC, also due to the presence of a plasticizer with a lower density, has a reduced density compared to PVC without modifiers. The introduction of ES into the PVC matrix to a lower degree, compared to CC, increases the density of the composites. In addition, the ES increases the porosity of the material compared to CC, which was an expected effect due to the porous structure of the filler and its significantly different particle sizes. Additional factors may be bubble errors formed in the plates of the pressed material related to the removal of air adsorbed by the ES structures or are related (to a small extent, as indicated by the TGA results) to the release of gaseous products formed from the decomposition of the organic part of the ES. The increase in porosity and the number of voids in the case of ES composites can have an unfavourable effect on the load transfer (act as crack initiators or act as inclusions) in the material and thus the mechanical properties, and their presence was evidenced in the surface images of the material samples after mechanical testing.

### 3.8. Mechanical Properties

**Figure 5:**
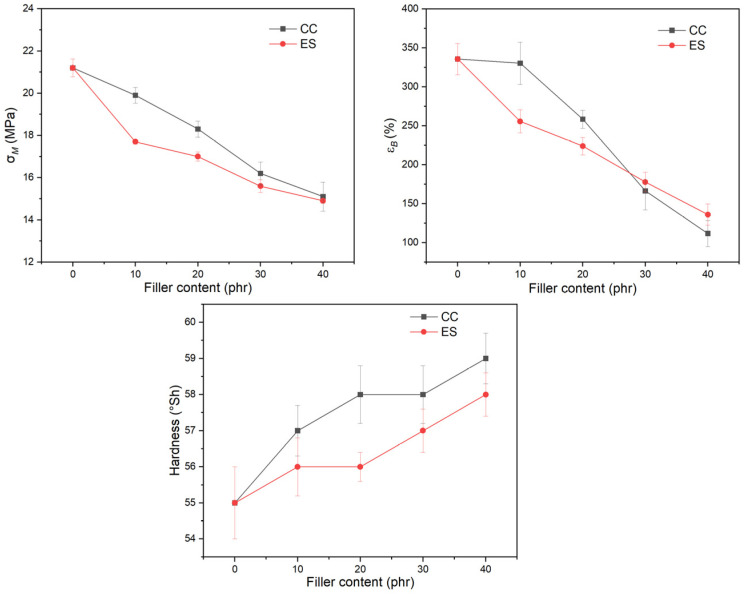
Characteristic values of mechanical properties: E_t_—modulus of elasticity; σ_M_—tensile strength, ε_B_—elongation at break (pPVC/CC—pPVC/ES).

Figure 5 presents the changes in tensile strength and elongation at break and the changes in Shore hardness as a function of type and filler content. 

The introduction of both types of filler results in lower σM and εB values, and with an increase in the amount of filler, these values are lower compared to the unfilled matrix (Figure 5). This effect is characteristic of the use of a high proportion of mineral fillers [82,83]. The surface modification of the fillers, as in the case of the CC used, is supposed to improve the adhesion between the matrix and filler [82,84], but this effect is too low to observe a strengthening effect. The fillers are characterized by a low aspect ratio, significantly limiting the transfer of stresses with increased deformation. Therefore, an increase in hardness values is observed and measured at low deformation despite other lower strength parameters. Compared to pPVC/CC, pPVC/ES has slightly lower mechanical property values, which may be due to both the fineness of the filler and the higher porosity of pPVC/ES. However, at 30 phr and 40 phr, the properties of the composites containing the applied filler are very similar, and the values are within the designated standard deviation of the measurement.

**Figure 6:**
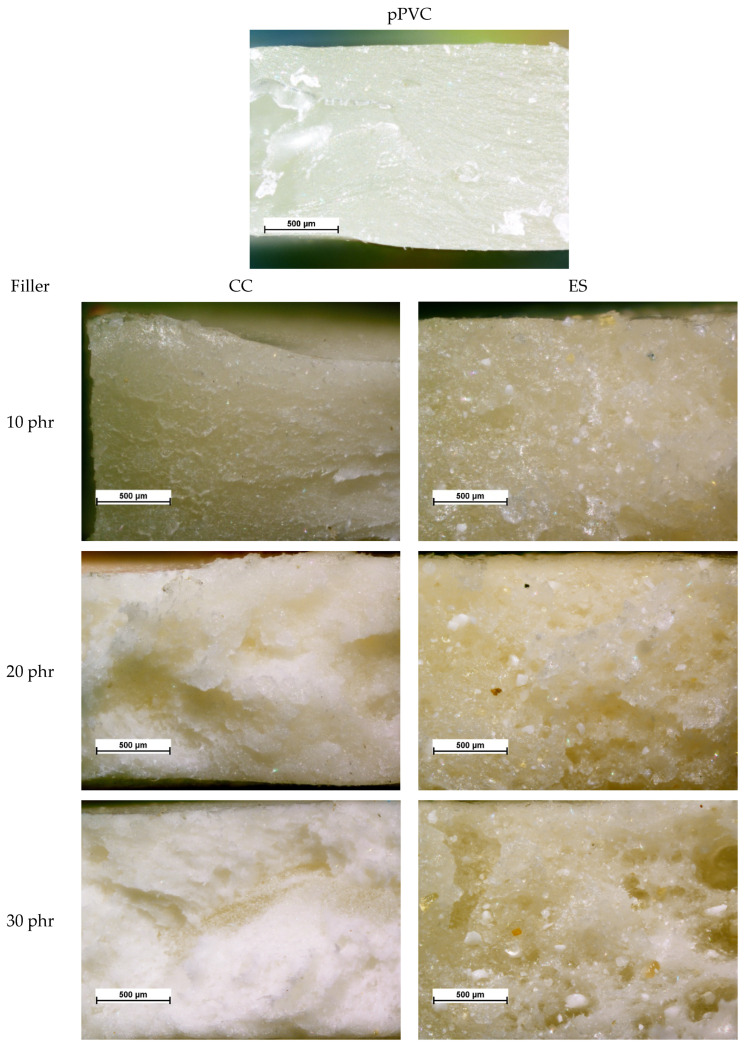

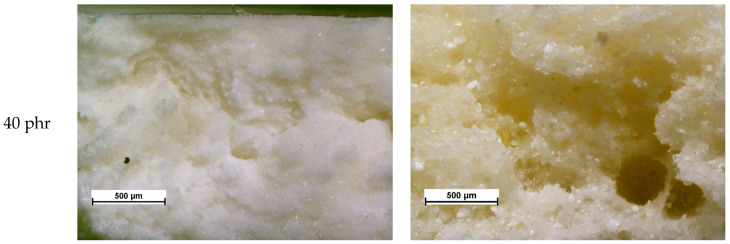
Images of fracture surfaces of materials after mechanical testing obtained by optical microscopy.

As already mentioned, the lower mechanical properties of pPVC/ES may be due to the different material structure. ES causes increased porosity, which was further confirmed by microscopic analysis of the broken surface of the sample after mechanical testing (Figure 6). The observed pores represent a discontinuity in the material, in which stresses are not transmitted. These stresses accumulate at the pores’ surface, resulting in high stress accumulation and the faster initiation of material breakage.

The fracture surface of pPVC is smoother, flatter, and more uniform than composites, but when the amount of filler in the matrix increases, the surface becomes uneven and rough. Unlike PVC/ES, no individual CC particles can be separated on optical microscopy images, and no visible pores or bubbles are observed. In contrast, the fracture surface of pPVC/ES composites is characterized by significant inhomogeneity, with large, irregularly shaped ES filler particles visible. As the share of ES in the PVC matrix increases, pores are also visible. This could be caused by the presence of air in the ES structures or the release of water from them. The voids which are present weaken the mechanical properties of the material.

### 3.9. Analysis of Thermal Properties

**Figure 7:**
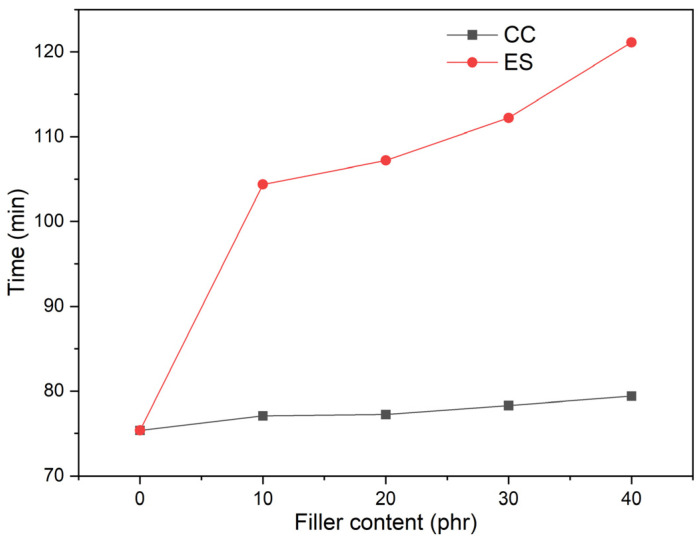
Time of thermal stability (t_ts_) as a function of the filler content in the pPVC/ES and pPVC/CC composites.

Regarding PVC matrix composites, it is extremely important to know the effect of fillers on thermal stability. Poor thermal stability and easy degradation limit the processing of this material. A commonly used method for assessing the time of thermal stability of PVC materials is the Congo red method, which allows the determination of the thermal stability of the material when heated at a constant temperature. The time of thermal stability (t_ts_) was determined as the time at which the indicator paper changes colour, i.e., the time after which gaseous HCl is emitted from the PVC material heated to 200 °C due to the decomposition of PVC macromolecules. Figure 7 shows the change in the time of thermal stability (t_ts_) depending on the composition of pPVC materials.

The value of t_ts_ of plasticized PVC was about 75 min. The EOS plasticizer used also influences the extension of the t_ts_ value, due to the presence of epoxy groups in the plasticizer structure, stabilizing the PVC macromolecules [85]. The pPVC/CC composites have similar t_ts_ value regardless of the amount of filler and are at a comparable level to the unfilled PVC matrix. Compared to PVC, the maximum increase in t_ts_ when using chalk was 5%. In contrast, PVC composites with ES, even at its lowest content (10 phr), are characterized by a significant increase in t_ts_, i.e., by 38%, and in the case of the composite with 40 phr ES, an increase of 61% compared to unmodified PVC. These results indicate that the ES obtained, compared to chalk, has a favourable and higher stabilizing effect, and its mechanism of action is based on the trapping and binding of HCl/Cl•- formed during the heating of PVC by CaCO_3_ and schematically follows the reaction:

[-CH_2_-CHCl-]_n_ + CaCO_3_ → [-CH = CH-]_n_ + CO_2_ + H_2_O + CaCl_2_

Other work has also observed a favourable effect of t_ts_ elongation due to the presence of chalk, especially when smaller particle sizes are used. This has been linked to the HCl capture effect being improved with an increased specific surface area of the chalk filler [86,87,88].

Calcium carbonate is characterized by a high heat capacity, as presented in the literature in the case of Omya CC, 880 J/kg K [89], for eggshell, 888 J/kg K [90], and in their ground form, 827–805 J/kg K [91]. Their presence in the polymer matrix can exert an insulating effect on the macromolecules of PVC during processing or thermal decomposition. These fillers absorb and evenly disperse heat across the entire volume of the material, which reduces the degradative effect of temperature on the PVC chains and thus extends the stability of the material. An additional factor can be the size of the filler particles, i.e., smaller particles with a higher specific surface area have a more beneficial effect on the dispersion and absorption of heat [86]. In the case of the fillers used, CC has much smaller and more uniform dimensions than the ES used. However, the observed effect of extending the thermal stability time of composites with ES is higher.

In the case of the ES, such a significant increase in the thermal stability of the PVC composites, despite the much higher dimensional values of the ES particles compared to chalk, can be related to the specific eggshell structure retained in the resulting filler. Namely, the porous sponge-like structure of the eggshell with its palisade-like structure of calcium carbonate crystals is a macroporous material that in the egg structure not only protects the embryo mechanically but also carries out gas exchange with the embryo. This porous sponge-like structure has been retained in the ES and can more effectively deactivate HCl/Cl• released from the PVC macromolecules. Similar conclusions were reached by Sharmeeni Murugan et al., stating that larger particle biobased fillers provide better thermal stability of the composites [40].

These conclusions are confirmed by the SEM images provided in our previous work [11]. This structure of the ES prolongs the thermal stability of PVC composites, especially in the first phase of macromolecule decomposition, when Cl radicals are formed and HCL is consequently released. ES acts as a radical scavenger. This mechanism involves catching and binding Cl radicals to the form of CaCl_2_, thus extending the time of the PVC macromolecules’ thermal stability.

Conducting TGA measurements in oxidizing atmospheres indirectly determined how a CC- or ES-modified PVC polymer matrix may perform during thermo-oxidative degradation, which occurs, for example, during processing. These tests provided insight into how fillers affect the initial decomposition temperature of materials. TGA studies in the air were carried out when evaluating the quality of thermal stabilizers of PVC [92].

**Figure 8:**
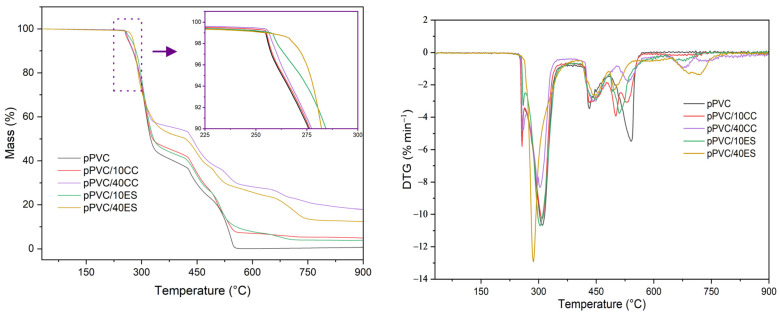
Example of TGA (upper plot) and DTG (lower plot) thermograms of pPVC and composites containing 10 and 40 phr of CC and ES.

Figure 8 presents the TGA and DTG thermogram of the pPVC matrix and the pPVC/40ES and pPVC/40CC composites. Table 4 summarizes the characteristic measured values.

The decomposition of PVC in an air atmosphere occurs in several stages, according to [85,86]; the onset of its decomposition, due to the oxidizing atmosphere, occurs at a lower temperature than in the case of measurements in a nitrogen atmosphere. The first stage of decomposition of approx. 57% by weight of the sample is related to the decomposition of the plasticized PVC matrix and the dechlorination of PVC macromolecules with the release of HCl gas. The evaporation and decomposition of the plasticizer used also occur in this temperature range. As a result, a polyene polymer structure is formed. This results in the formation of a polyene polymer structure. At this stage, the absorption and bonding of the released HCl by the CaCO_3_ particles can occur and thus shift the decomposition temperature towards higher values. The second stage of decomposition in the temperature range of approx. 400–550 °C is associated with the decomposition of the cross-linked polyene structure with the formation of water and CO_2_ [78,93,94].

In the case of plasticized PVC composites with CC and ES, the TGA thermograms are basically similar to that of pPVC. In the first stage, where the dehydrochlorination of the PVC macromolecules occurs, there also occurs a reaction between HCl or the Cl radical and CaCO_3_, which results in the release of CO_2_ and the formation of CaCl_2_ as well [93,94]. In the temperature range of 600–800 °C, the TGA curves of the composites include a decomposition step associated with the thermal degradation of carbonate fillers with the evaporation of CO_2_ [93].

Mineral fillers such as chalk or gypsum [95] can react with secreted HCl or Cl radicals from the PVC macromolecule and thus effectively bind and deactivate them [11]. This action protects the macromolecules from their catalytic action and from the further degradation of the PVC macromolecules.

**Figure 9:**
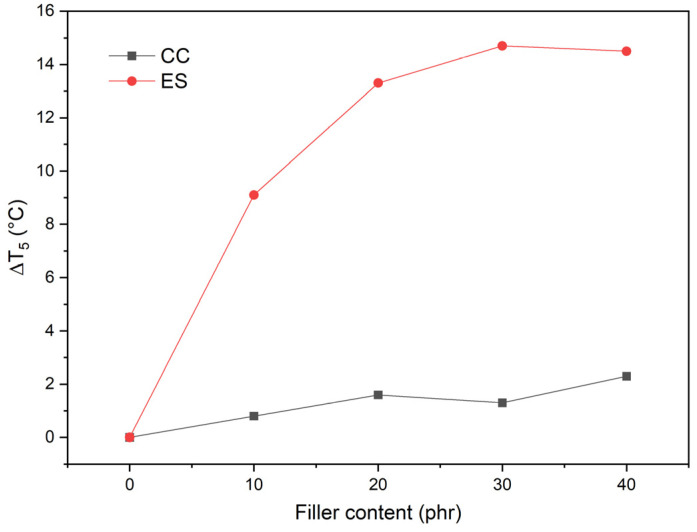
Variation in ΔT_5_ values of pPVC composites as a function of type and filler content in the matrix.

Substances with HCl/Cl scavenging activity must be effective in the first stage of PVC material degradation, i.e., in the initial stage, to minimize the catalytic effect of HCl/Cl on macromolecules. Analyzing the decomposition of the materials in the initial phase, the temperature T5 was taken as the start of the decomposition, i.e., the temperature at which 5% of the sample mass has decomposed, and the T_5_ values obtained, higher than in the case of pPVC, indicate the positive protective effect of the filler. Figure 9 summarizes the differences in T_5_ values between the unfilled matrix and the composites (ΔT_5_).

Adding chalk to the plasticized PVC matrix has a negligible effect on the increase in ΔT_5_ values (increase in the range 0.8–2.3 °C), which increased slightly depending on the amount of CC in the matrix. Compared to pPVC, the most significant ΔT_5_ values (14.5 °C) were obtained for composites with the highest ES content (30 and 40 phr).

Based on the analysis of the ΔT_5_ values, it is clear that the ES has a more substantial effect on extending the thermal stability of the composite. Despite its large size, compared to CC, this filler is characterized by high porosity with a three-level hierarchy: nano-scale pores, submicron-scale pores (200–400 nm), and micro-scale [33], which was retained during filler preparation. This spongy ES structure interacts more effectively with the forming Cl radicals from the decomposition of PVC macromolecules, deactivating them in the first stage of their decomposition, which translates into an increase in thermal stability. This effect was used for the utilization of PVC during its dechlorination in the presence of eggshells [90], which can also be a valuable source of filler for PVC matrix composites [37,40], showing a stabilizing effect as a secondary stabilizer scavenging the HCl/Cl radical generated [96]. This effect was used to utilize PVC during its dechlorination in the presence of eggshells, which can also be a source of a valuable filler for PVC matrix composites, showing a stabilizing effect as a secondary stabilizer by scavenging the HCl/Cl radicals generated.

Fillers with a stabilizing effect support the primary stabilizers in protecting the PVC macromolecules from degradation. Thus, a filler derived from ES can perform this role. In addition, ES is non-toxic, derived from renewable sources, and environmentally friendly.

## 4. Summary

Waste eggshells can be a valuable source of filler for poly(vinyl chloride) materials. ES-modified PVC composites significantly improved thermal stability compared to commonly used modified chalk.

The plasticized PVC/ES composites showed slightly higher processing friction than CC. The processing properties of pPVC/CC, analyzed as torque, are more favourable than using ES. The torque value after 10 min of kneading is 21–25% higher in PVC composites with 20 phr and 40 phr of ES, while about 7 °C reduces the temperature of the plasticized mixture. This is due to the absence of additional ES surface modifiers. In PVC processing technology, this is not a significant limitation, as the use of processing property modifiers is common [27,45].

PVC/ES at 10 phr and 20 phr has noticeably lower mechanical properties compared to analogous composites with PVC/CC. At higher concentrations of both types of fillers, the differences in mechanical properties are already slight and within the standard deviation. PVC/ES composites, especially at low filler concentrations, will require work on more efficient mechanical load transfer in further application considerations, such as compatibilizers, silanes, or other improvement methods.

The essential and most important benefit of using ES to modify PVC is the considerable improvement in the thermal stability of this material. The benefit of the proposed material is the significantly increased thermal resistance of the PVC matrix composites, especially in the initial degradation phase, measured as an increase in thermal stability time and the temperature of the onset of decomposition. This results from deactivating the Cl• radicals or evolved HCl with an autocatalytic effect formed at high temperatures. This intensifies the filler’s stabilizing impact on the material’s thermal protection by simultaneously insulating, absorbing heat, and scavenging HCl/CL•. This is particularly significant in the case of PVC matrix materials, especially in terms of their multi-stage processing or recycling. Additionally, the increased ability of ES to capture the resulting HCL can be used to dispose of PVC materials during their incineration. In application considerations, given the failure to improve the mechanical properties of PVC/ES composites, it would be appropriate to consider using a small amount of ES as an additive to improve thermal stability in other PVC matrix composites.

The idea of a plasticized PVC matrix material using a plasticizer of natural origin and a biofilter from waste sources corresponds to sustainable and circular economy issues. Eggshells are a promising alternative to commercial CC sources, but replacing the commercial CaCO_3_ filler source would be uneconomical, starting from the availability of the eggshell waste, its selective collection, or its separation from the waste stream, e.g., employing collection points at industrial processing sites, food factories, or chicken farms.

## Figures and Tables

**Table 1:** Plastographometric analysis.

Material	Kneading Time					
	2 min		5 min		10 min	
	M (Nm)	T (°C)	M (Nm)	T (°C)	M (Nm)	T (°C)
pPVC	18.4 (0.9)	162.0 (0.3)	14.5 (0.5)	171.4 (0.2)	12.2 (0.3)	174.3 (0.2)
pPVC/10ES	20.1 (1.0)	150.1 (1.8)	18.5 (1.2)	161.1 (0.9)	16.5 (0.8)	164.5 (0.4)
pPVC/20ES	17.6 (1.3)	154.3 (1.0)	16.1 (0.6)	163.3 (0.8)	14.4 (0.2)	166.8 (0.3)
pPVC/30ES	18.4 (0.7)	155.1 (1.8)	16.8 (0.5)	163.9 (0.3)	14.6 (0.2)	167.5 (0.4)
pPVC/40ES	19.3 (1.0)	155.5 (1.1)	17.1 (0.5)	164.0 (0.3)	14.7 (0.3)	167.4 (0.2)
pPVC/10CC	16.6 (0.8)	163.0 (0.6)	13.8 (0.3)	171.8 (0.3)	11.1 (0.1)	174.7 (0.1)
pPVC/20CC	16.1 (1.1)	160.2 (0.4)	13.1 (0.3)	170.0 (0.2)	11.5 (0.2)	173.4 (0.2)
pPVC/30CC	16.6 (0.6)	161.5 (0.3)	13.0 (0.4)	170.6 (0.3)	11.6 (0.1)	174.1 (0.1)
pPVC/40CC	17.5 (0.5)	161.4 (0.2)	13.5 (0.3)	171.1 (0.4)	12.1 (0.2)	174.5 (0.1)

**Table 3:** Results of density and porosity.

Sample	Density (g/cm^3^)	Volume Fraction of Filler	Porosity (%)
CC	2.668	-	-
ES	2.266	-	-
pPVC	1.284	-	-
pPVC/10ES	1.299	0.060	3.29
pPVC/20ES	1.327	0.128	5.83
pPVC/30ES	1.387	0.204	6.59
pPVC/40ES	1.428	0.292	9.11
pPVC/10CC	1.327	0.051	0.61
pPVC/20CC	1.372	0.110	1.48
pPVC/30CC	1.424	0.178	2.38
pPVC/40CC	1.487	0.257	3.23

**Table 4:** Results of thermogravimetric analysis.

Sample	The Temperature of Weight Loss			Temperature of the Maximal Degradation Rate (T_DTG_) (°C)	Residual Mass at 900 °C (%)
	T_1_ (°C)	T_5_ (°C)	T_50_ (°C)		
pPVC	254.7	262.6	323.4	309.8	0.2
pPVC/10ES	257.4	271.7	332.7	303.5	3.8
pPVC/20ES	259.3	275.9	342.1	292.2	8.7
pPVC/30ES	262.5	277.3	371.5	289.3	9.4
pPVC/40ES	255.1	277.1	418.9	286.2	12.4
pPVC/10CC	255.3	263.4	329.8	309.2	5.0
pPVC/20CC	255.9	264.2	355.0	307.1	9.3
pPVC/30CC	255.9	263.9	423.1	304.3	13.6
pPVC/40CC	256.2	264.9	437.5	303.4	17.9

## Data Availability

The raw data supporting the conclusions of this article will be made available by the authors on request.

## Abbreviations

The list of most essential abbreviations:

CC	Commercial Calcium Carbonate
CIE L*a*b*	Commission Internationale De l’Eclairage (CIE) By Measuring The L*a*b* Colour
Cl•	Chlorine Radical
DTG	Derivative Thermogravimetry
EOS	Epoxidised Soybean Oil
ES	Filler From Eggshell Waste
E_t_	Modulus Of Elasticity
FTIR	Fourier-Transform Infrared Spectroscopy
GCC	Ground Calcium Carbonate
HCL	Hydrogen Chloride
M	Torque
PCC	Precipitated Calcium Carbonate
phr	Part Per Hundred
pPVC	Plasticized PVC
PVC	Poly(Vinyl Chloride)
T	Temperature
TGA	Thermogravimetric Analysis
t_ts_	Time of Thermal Stability
XRD	X-ray Diffraction
ΔE	Total Colour Difference Parameter
ΔT_5_	Differences In T_5_ Values Between The Unfilled Matrix And The Composites
ε_B_	Elongation At Brake
σ_M_	Tensile Strength
CC	Commercial Calcium Carbonate
